# Effect of oxidative aging of biochar on relative distribution of competitive adsorption mechanism of Cd^2+^ and Pb^2+^

**DOI:** 10.1038/s41598-022-15494-y

**Published:** 2022-07-04

**Authors:** Zhe Wang, Chengxin Geng, Yuan Bian, Guangyu Zhang, Chunli Zheng, Chunjiang An

**Affiliations:** 1 School of Energy and Environment, Inner Mongolia University of Science and Technology, Baotou, 014010 China; 2 Inner Mongolia Engineering Research Center of Evaluation and Restoration in the Mining Ecological Environment, Inner Mongolia University of Science and Technology, Baotou, 014010 China; 3 Department of Building, Civil and Environmental Engineering, Concordia University, Montreal, QC H3G 1M8 Canada

**Keywords:** Biochemistry, Environmental sciences

## Abstract

In this study, aged biochar (CCB350 and CCB650) were obtained from pyrolysis of corn stalk biochar (CB350 and CB650) at the degree of 350 °C and 650 °C by artificial oxidation with hydrogen peroxide (H_2_O_2_). Also, the mechanism of Pb^2+^ and Cd^2+^ on fresh and aged biochars was analyzed qualitatively and quantitatively by batch adsorption experiments combined with characterization. The adsorption isotherm results showed that aging treatment decreased the adsorption capacity of Pb^2+^ and Cd^2+^ and inhibited the competitive adsorption behavior of heavy metals. In the single-metal system, precipitation and cation exchange were considered as the main adsorption mechanisms for CB350 and CB650, with a ratio of 40.07–48.23% and 38.04–57.19%, respectively. Competition between Pb^2+^ and Cd^2+^ increased the relative contribution of mineral precipitation, but decreased the contribution of cation exchange mechanism. Aging resulted in the rise of the contribution of surface complexation to the adsorption of Pb^2+^ and Cd^2+^ on biochars, especially in low-temperature biochars, but weakened the contribution of mineral precipitation to the adsorption. Further, the contribution of other adsorption mechanisms was significantly enhanced for high-temperature aged biochars. These results are important to evaluate its long-term application prospects in the natural environment.

## Introduction

The problem of compound pollution of heavy metals in soil is a matter of widespread concern, which threatens both the environment and human health^1,2^. Cadmium and lead are elements that occur widely in contaminated soils and they are also classified as priority pollutants in many countries^3,4^. The combined contamination of the two heavy metals produces greater ecotoxicity than either one of them^5^. Therefore, it is important to explore suitable technologies to remediate the metals polluted soil. However, the interaction of multiple heavy metals limits the effective application of many remediation techniques^6^. Biochar is considered an eco-friendly, reliable, and low-cost adsorbent, which is widely used in the remediation of contaminated soil^7–11,12^. There are many resources for corn straw in northern China, but the harmless treatment of corn straw has always been a difficult problem to solve. Therefore, biochars produced from corn straw can contribute to environmental cleanup practices and waste resource recovery efforts.

Heavy metal adsorption depends not only on biochar characteristics but also on the competitive behaviors of the metals. Most previous studies have mainly focused on changes in competing sorption capacity and selective sequences of heavy metals using different biochars. For instance, Ni et al.^13^ evaluated the competing sorption by biochar from anaerobically digested sludge in an aqueous solution. Abdin et al.^14^ concentrated on the removal of the single and competing metals (Cd^2+^, Pb^2+^, Zn^2+^ and Cu^2+^) from aqueous solutions by mesocosms and fish bone biochar. Han et al.^15^ studied Pb^2+^ adsorption by three typical types of biochars (cattle manures, rice husks, and bamboo biochars) under the elevated competition of Cd^2+^ and Al^3+^. These studies illustrate that Pb^2+^ has a higher affinity than Cd^2+^, thus showing a better competitive advantage in the coexistence system. However, the relationship between competition and the adsorption mechanism of Cd^2+^ and Pb^2+^ has not attracted much attention.

The adsorption mechanism determines the removal rate of these metals. Therefore, studying on competitive adsorption mechanism of heavy metals plays a significant role^16^. The mechanisms of heavy metals adsorbed on biochars under single metal adsorption conditions involve precipitation or co-precipitation^17^, ion exchange^18^, complexation^19,20^, cation-π interaction^21,22^, and physical adsorption^23^. Although previous studies have investigated the dominant mechanisms of the metals adsorbed on biochars in a single-metal solution system, relevant information about the impact of competition on the change in the main adsorption mechanism is limited, especially those quantifying the effects.

Biochar properties are stable in the short term, but they may undergo a long-term changing process when added into soil, that is, aging^10,11^. Various environmental factors, such as freeze–thaw cycles^24^, wetting–drying cycles^25^, photochemical degradation^26^, and mild oxidation^27^ will lead to significant impact on the biochar, including its pH value, specific surface area (SSA), cation exchange capacity, oxygen-containing functional groups, elemental composition, ash content, and surface morphology^28–32^. However, it is very difficult to really learn about the features of biochar during the long natural aging process. Therefore, various artificial aging methods are often used to predict changes in the physiochemical properties and adsorption capacity of biochar^33^. The application of hydrogen peroxide (H_2_O_2_) can simulate the aging process of natural oxidation of biochar^34^. The competitive adsorption mechanism of Cd^2+^ and Pb^2+^ largely depend on biochars’ properties and the adsorbed solution, such as biochar surface characteristics, solution pH, and concentration of ions in solution, which are affected by aging process. However, qualitative and quantitative studies on the competitive adsorption mechanisms of Cd^2+^ and Pb^2+^ on aged biochar are not yet extensive and in-depth. Therefore, we assumed that the proportion of mechanisms for Cd^2+^ and Pb^2+^ adsorbed on biochars varied in co-existed adsorption conditions and biochar aging affected the contribution of adsorption mechanisms to heavy metals.

In this study, biochars were obtained by pyrolysis of corn straw at 350 °C and 650 °C and simulated aging process with H_2_O_2_, and the surface properties of fresh and aged biochar were characterized. Moreover, the Cd^2+^ and Pb^2+^ for kinetics and isotherms on fresh biochar (CB350 and CB650) and aged biochar (CCB350 and CCB650) were examined. In short, this paper has the following objectives. First, the effect of biochar aging on adsorption characteristics of Cd^2+^ and Pb^2+^ in single and binary adsorption conditions was clarified. Secondly, the effects of aging on the quantitative distributions of Cd^2+^ and Pb^2+^ adsorption mechanisms in single and binary conditions were evaluated.

## Materials and methods

### Preparation of biochars

Samples of corn straw used to produce biochar were collected from Baotou, Inner Mongolia, China. The preparation method for fresh biochar (CB350 and CB650) is provided in the supplementary materials.

Aged boichars were prepared as described by Nie et al.^35^. Briefly, the fresh biochars (10 g) and 20% H_2_O_2_ (200 mL) were mixed with the solid–liquid ratio of 1:20 (w/v) in a plastic bottle of 250 mL, and the samples were presented in a reciprocating shaker at 24 h room temperature. In the process of oxidation, the bottles were opened repeatedly to release air. After aging, the samples were filtered by centrifugation and rinsed several times. And the residual H_2_O_2_ was removed with distilled water. The aged biochars were dried at 80 °C and labeled as CCB350 and CCB650, respectively. The surface characteristics of the biochars were also analyzed using SEM–EDS, FTIR, XPS, XRD, and BET. The ash content, pH, surface zeta potential and total C, H, O, N and S contents of biochars were measured. The details were described in the supplementary materials.

### Batch adsorption experiments

A certain amount of Cd(NO_3_)_2_·4H_2_O and Pb(NO_3_)_2_ were dissolved in the background solution (0.01 mol/L NaNO_3_) to prepare Cd^2+^ (1000 mg/L) and Pb^2+^ (1000 mg/L).

0.1 g biochar (CB350, CB650, CCB350 and CCB650) was mixed with 50 mL Cd^2+^ or Pb^2+^ solution for adsorption isotherm experiment. The mass ratio of Cd^2+^ to Pb^2+^ was chosen as 1:1 in the binary system. The initial pH of the solution was adjusted to 5.0 ± 0.1 by 0.1 mol/L NaOH or 0.1 mol/L HNO_3_. This pH ensured the maximum adsorption capacity of biochar while avoiding the effect of Pb^2+^ or Cd^2+^ adsorption due to precipitation. We added 0.01 mol/L of NaNO_3_ to all the solutions as background electrolyte. The concentration of Cd^2+^ and Pb^2+^ in both the single and binary metal systems range from 0 to 200 mg/L. The biochar and different concentrations of Cd^2+^ or Pb^2+^ solutions were then shaken in an HY-4 speed-controlled multipurpose shaker (Jintan Ronghua Instrument Manufacturing Company, Jinhua, China) at 200 rpm and 25 ± 1 °C for 2880 min.

As for adsorption kinetics, 0.1 g of biochars (CB350, CB650, CCB350, and CCB650) were mixed with 50 mL of 100 mg/L for single Cd^2+^, single Pb^2+^ and binary Cd^2+^ and Pb^2+^ at room temperature, and shaken at 200 rpm for 2880 min.

Finally, the suspension was centrifuged at 4000 rpm for 20 min and filtered by a 0.45 μm microporous filter. The concentrations of Cd^2+^ and Pb^2+^ in the filtrate were determined by flame atomic absorption spectrophotometry (Ice 3500, Thermo Fisher Scientific, USA). The adsorption amount of heavy metals on biochar (*Q*_e_) was calculated as follows:

1 $${\text{Q}}_{\text{e}}=\frac{\left({\text{C}}_{0}-{\text{C}}_{\text{e}}\right){\text{V}}}{\text{m}}$$

where *Q*_e_ (mg/g) is the adsorption capacity, *C*_0_ (mg/L) and *C*_e_ (mg/L) are the initial and equilibrium concentration, respectively. *V* (L) is the volume of mixed solution, and *m* (g) is the dry mass of biochar.

The adsorption isotherms were simulated using Langmuir, Freundlich and Langmuir–Freundlich models, and the adsorption kinetics were fitted using Lagergren’s pseudo-first-order (PFO), pseudo-second-order (PSO) and intra-particle diffusion (IPD) kinetic equations (see Supplementary Material).

Inherent minerals were removed by rinsing with 1.0 mol/L HCl to obtain demineralized biochars. And the untreated and demineralized biochars were mixed with solutions of Pb^2+^ and Cd^2+^ 200 mg/L. After adsorption, the solution concentration was measured. The fresh biochar was then mixed with 0.01 mol/L NaNO_3_ (blank) and metal salt solution, respectively. The contribution of cation exchange can be estimated by the difference of K^+^, Ca^2+^, Na^+^ and Mg^2+^ cations released into the solution after biochar adsorption of heavy metals and the number of cations released in the blank solution. To determine the role of complexation mechanisms, the difference of pH were calculated before and after adsorption. The reduced adsorption capacities of Cd^2+^ and Pb^2+^ on the biochars before and after demineralization attributed to the mineral precipitation. Flame atomic absorption spectroscopy was used to analyze the Na^+^, Mg^2+^, K^+^, Ca^2+^, Cd^2+^ and Pb^2+^ concentrations in the filtrates. Biochars carried with and without Cd^2+^, Pb^2+^ and the Cd^2+^/Pb^2+^ were accumulated for the measurements of SEM–EDS, XRD, and FTIR.

### Quantitative contribution of adsorption mechanisms

The adsorption capacities of the fresh and aged biochars for Cd^2+^, Pb^2+^ and the Cd^2+^/Pb^2+^ were attributed to the following four fractions: (1) complexation between surface oxygen-containing functional groups (*Q*_com_), (2) mineral co-precipitation (*Q*_pre_), (3) cation exchange (*Q*_ce_), and (4) other potential mechanisms (*Q*_oth_)^36^. The adsorption capacity of each adsorption mechanism was calculated as follows:

1. Complexation of oxygen functional groups (*Q*_com_): Due to the adsorbing Cd^2+^ and Pb^2+^ by demineralized biochar, the carboxyl and hydroxyl groups of the biochar can release H^+^ through ion exchange, which in turn resulting in the changing of the pH. Therefore, the drop in pH was used as an indicator of H^+^ release and the amount of Cd^2+^ or Pb^2+^ adsorbed due to *Q*_com_^37^.
2. Metal adsorption resulting from cation exchange (*Q*_ce_): The release amount of Na^+^, Mg^2+^, K^+^, Ca^2+^ was calculated by the net release amount of them by the comparison after biochar adsorption^38^ (Eq. (2)):
  2 $$ Q_{{{\text{ce}}}} = Q_{{\text{K}}} + Q_{{{\text{Na}}}} + Q_{{{\text{Mg}}}} + Q_{{{\text{Ca}}}} $$
  They are the net release amounts of K^+^, Ca^2+^, Na^+^ and Mg^2+^ during adsorption process, respectively.
3. Mineral co-precipitation (*Q*_pre_): The demineralized biochar obtained after acid washing does not cause vital changing of the content of surface oxygen functional groups on its surface^39^. The adsorption capacity generated by mineral precipitation can therefore be measured as the difference of Cd^2+^ or Pb^2+^ by virgin biochar (fresh and aged biochar) and demineralized biochar. In turn, the adsorption due to mineral components was divided into ion exchange (*Q*_ce_) and precipitation (*Q*_pre_). Therefore, it could be calculated as follows^40^ (Eqs. (3) and (4)):
  3 $$ Q_{{{\text{pre}}}} = \, Q_{{\text{m}}} {-} \, Q_{{{\text{ce}}}} $$
  4 $$ Q_{{\text{m}}} = \, Q_{{\text{t}}} {-} \, Q_{{\text{d}}} $$
  *Q*_m_ (mg/g) is the amount of Cd^2+^ or Pb^2+^ absorbed by biochar in the interaction with minerals, *Q*_t_ (mg/g) is the total amount of metal absorbed by biochar, and *Q*_d_ (mg/g) is the amount of Cd^2+^ as well as Pb^2+^ absorbed by demineralized biochar.
4. Other mechanisms (*Q*_oth_): Except for the above mechanisms of adsorption, other mechanisms (e.g., cation-π interaction, electrostatic gravitational force, and physical adsorption) cannot be neglected as well. Therefore, the *Q*_oth_ can be obtained as follows^41^ (Eq. (5)):
  5 $$ Q_{{{\text{oth}}}} = \, Q_{{\text{d}}} {-}Q_{{{\text{com}}}} $$

The contribution ratios of other mechanisms to the adsorption of metal are described below. The ratios of *Q*_com_/*Q*_t_, *Q*_ce_/*Q*_t_, *Q*_pre_/*Q*_t_, and *Q*_oth_/*Q*_t_ can define the contribution percentages of different mechanisms to metal adsorption.

### Statistical analysis

The experimental results were an average obtained from three adsorption experiments. According to Origin 9.0, we can obtain adsorption kinetics and isotherm data for Cd^2+^ and Pb^2+^ (Origin Lab, USA). In addition, mineral components of aged and fresh biochars were analyzed using Jade 6.5.

### Ethical approval

This study did not involve human or animal research and did not require ethical approval.

## Results and discussion

### Changes in biochar properties with aging

Table S1 shows the main physicochemical properties of fresh and old biochar. As the pyrolysis temperature increased, for all the biochars, the pH and ash content also increased. The biomass material were mainly the evaporation of water and the volatilization of low boiling point substances at a low temperature. The high boiling point materials in biochars volatilized and inorganic mineral components increased with the temperature rising. As a result, the pH and ash of biochars were higher at higher pyrolysis temperatures^42^. After the chemical aging treatment, the pH and ash content showed a downward trend, especially for CCB350, which even became acidic. Oxidation reaction of hydrogen peroxide on the biochar surface may cause a rise in the functional groups of acidic oxygen, leading to a fall in the pH of the biochars^23^.

The SSA and SEM images are illustrated in Table S1 and Fig. S1. The SSA of both fresh and aged biochar increased significantly with the pyrolysis temperature. The SSA of CB650 and CCB650 increased by 99.23% and 99.08%, respectively, compared with CB350 and CCB350, respectively. The increase of temperature removed more volatile matter, which led to the formation of many micropores and even nanostructures, and thus increased the biochar surface area^43^. SEM results showed that CB350 and CB650 exhibited a porous fibrous structure, and the pore structure of the biochar became smoother and clearer after ageing treatment. Furthermore, the SSA of CB350 and CB650 also increased by 43.59% and 32.12% after chemical oxidation, which could be illustrated by the finding that the oxidation process was usually accompanied by a more intense decomposition and consumption of organic matter, which led to the expansion of biochar pores and the creation of more pore structures^44^. Further, the reduction of impurities washed away by the water bath heating process led the pore structures to become smoother^45^, which was confirmed by the results of SEM images. The similar finding was evaluated by Mia et al.^46^, believing that the SSA of biochar rose after the oxidation of hydrogen peroxide.

With the exception of CB650, when the pyrolysis temperature was increased, the C content of the biochar rose, while the O, H and N content were on the decrease. The contents of O were higher for the aged biochars than the fresh biochars, but the contents of C were lower. The changing of C and O contents indicated that aging may decompose unstable aliphatic carbon in biochar and introduce more oxygen functional groups, resulting in a fall in C and an obvious rise in O^47^. For CB650, the C content increased by 7.32% after chemical oxidation treatment, which may be due to the mass loss caused by the activation of own oxygen and ash outweighing the oxidation loss of more resistant matrix carbon^48^. In general, O/C, H/C and (O + N)/C are used as evaluation indexes of hydrophilicity, aromatics, and polarity of biochar, respectively^49^. After chemical oxidation, the O/C values of CB350 and CB650 increased by 86.27% and 38.89%, respectively, indicating that chemical oxidation enhanced the hydrophilicity of biochar. At the same temperature, the low (O + N)/C meant that there were relatively fewer polar functional groups on the surface of fresh biochar.

Surface functional groups and crystalline minerals in the biochar were characterized by FTIR and XRD, as shown in Fig. S2. FTIR results showed that CCB350 had stronger intensity of bands at 3414 cm^−1^ and 1690 cm^−1^ compared to CB350, indicating that CCB350 exhibited a higher abundance of –OH and –COOH. CCB650 showed a more significant enhancement of the C–O–C stretching vibration peak at 1021 cm^−1^ than CB650. In summary, the aging treatment induced more O-containing functional groups. The XRD pattern showed that the main crystalline form on the surface of fresh biochar was KCl, and the KCl peak weakened or even disappeared after chemical aging, probably because of the high solubility of KCl in water.

In order to analyze the effects of aging treatments on the oxygen-containing functional components on the surfaces of biochar, XPS analysis was further performed. The fitted spectra of fresh and aged biochars are shown in Fig. S3. The main C1s peaks of biochars were divided into four peaks: C–C, C–O, C=O, and O=C–O^19^. In fresh biochars, the content of C–C increased from 59.04 to 70.24% with the increase of pyrolytic temperature. This indicated that the increase of pyrolytic temperature could reduce the content of oxygen-containing functional groups on the surface of biochar. After chemical oxidation aging, the C-O contents of CB350 and CB650 increased by 0.32% and 5.02%, respectively. The C=O contents of CB350 and CB650 increased from 6.24% and 14.62% to 14.91% and 17.47%, respectively. Overall, biochars exhibited an increasing trend for the oxygenated functional groups.

### Effects of aging on the adsorption isotherms of Pb^2+^ and Cd^2+^ by biochars

**Figure 1:**
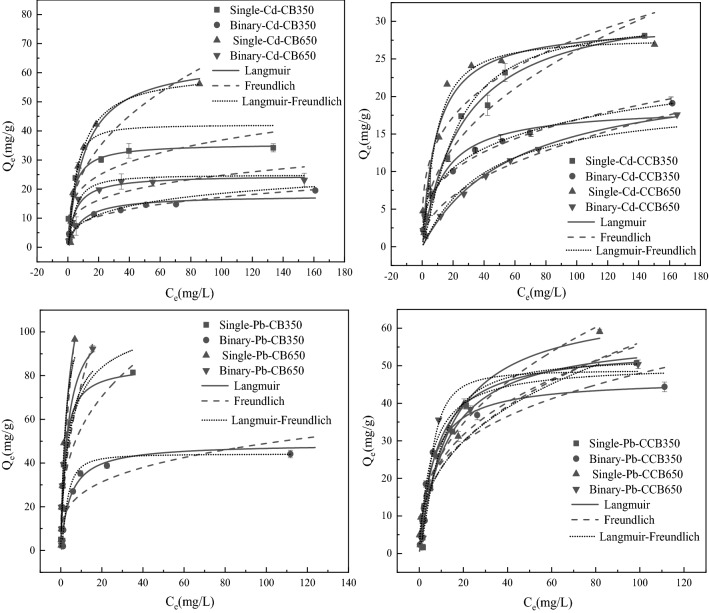
Adsorption isotherms of Pb^2+^ and Cd^2+^ on the fresh and aged biochars in the single and binary systems.

The adsorption isotherms of Pb^2+^ and Cd^2+^ on fresh and aged biochars are shown in Fig. 1. The adsorption abilities of them on all biochars increased rapidly with increasing heavy metal concentrations at the initial stage. After that, it inclined to increase slowly and gradually saturate, except for the Pb^2+^ adsorption isotherms on CB650, indicating that the surface absorbing sites on CB650 were not totally covered by Pb^2+^. In the binary metal system, the presence of coexisting ions significantly altered the adsorption isotherms of them. The adsorption isotherms in binary metal systems had a shorter initial linear part and could reach equilibrium at lower concentrations. Therefore, this indicated that the presence of competitive ions achieved the effect of completely occupying the adsorption sites of biochar^50^. Aging treatment also had an effect on the sorption isotherms of Pb^2+^ and Cd^2+^. The adsorption capacity of aged biochar to heavy metals was lower than that of fresh biochar.

The adsorption isotherms were suitable for the Langmuire, Freundlich and Langmuir–Freundlich models in both metal systems. The fitted parameter values are shown in Table S2. All fresh biochar adsorption isotherms of Pb^2+^ and Cd^2+^ were better fitted by the Langmuire model (*R*^2^ = 0.9490–0.9822). Most of the adsorption isotherms for the aged biochars were better fitted by Langmuir–Freundlich model, which suggested that the adsorption of metal ions on biochar after aging transformed from uniform adsorption to heterogeneous surface monolayer adsorption^23^. That is, it was close to the Freundlich isotherm at low concentrations of metal ions, while it described monolayer adsorption in a similar way to the Langmuir isotherm at high concentrations [^51^]. The *Q*_m_ of CB350 and CB650 for Pb^2+^ absorption using the Langmuir model were 84.91 mg/g and 149.98 mg/g, better than that of Cd^2+^ on CB350 and CB650 (35.66 mg/g and 65.43 mg/g, respectively). Competition affected the adsorption on biochars, the *Q*_m_ values of Cd^2+^ on CB350, CCB350, CB650, and CCB650 decreased by 49.99%, 43.97%, 62.11%, and 20.66%, respectively. The *Q*_m_ values of Pb^2+^ on CB350, CCB350, CB650, and CCB650 decreased by 42.45%, 20.73%, 25.91%, and 18.33%, respectively. The decrease in *Q*_m_ values was greater for Cd^2+^ adsorption than for Pb^2+^ under competitive conditions. Therefore, compared to Cd^2+^, Pb^2+^ was more competitive.

Moreover, the ratio of a*(*Q*_e_ of Pb^2+^ or Cd^2+^ in coexisting systems)/a (*Q*_e_ of Pb^2+^ or Cd^2+^ in single metal system) could also be used to indicate the degree of reduction in the adsorption capacity of Pb^2+^ or Cd^2+^ because of the competition for sites of adsorption in coexisting systems^52,53^. We evaluated the a*(Pb^2+^)/a (Pb^2+^) ratios for CB350, CCB350, CB650, and CCB650 were 0.54, 0.75, 0.95, and 0.85, respectively, while the a*(Cd^2+^)/a(Cd^2+^) ratios were 0.57, 0.68, 0.41, and 0.66, respectively. The a*(Pb^2+^)/a(Pb^2+^) ratios were greater than the a*(Cd^2+^)/a(Cd^2+^) ratios for all biochars except CB350, showing that the adsorption of Cd^2+^ was more influenced by Pb^2+^. In particular, the ratio a*(Pb^2+^)/a(Pb^2+^) (0.95) was close to 1, indicating that the existence of Cd^2+^ had limited impact on the adsorption of Pb^2+^ on CB650. This may be due to the following reasons: (1) compared to Cd^2+^ (4.26 Å), Pb^2+^ has a tinier hydrated radius (4.01 Å), and (2) Pb^2+^ has a higher electronegativity (2.33) than Cd^2+^ (1.69)^13^. Therefore, Pb^2+^ has stronger affinity for most functional groups and occupies more internal complexing sites in the coexistence system^54^.

After aging, the ratio of a*(Pb^2+^)/a(Pb^2+^) for CCB650 decreased to 0.85, while the ratio of a*(Cd^2+^)/a(Cd^2+^) increased to 0.66, indicating that aging inhibited the competitive adsorption of Pb^2+^ and Cd^2+^ by CB650. The competitive adsorption performance of low-temperature biochar was completely different from high-temperature biochar. The values of a*(Cd^2+^)/a(Cd^2+^) and a*(Pb^2+^)/a(Pb^2+^) on the fresh biochar (CB350) were close, while the a*(Cd^2+^)/a(Cd^2+^) and a*(Pb^2+^)/a(Pb^2+^) ratios of CCB350 grew to similar levels after aging. The above results indicated that there was no significant competitive adsorption in fresh and aged low temperature biochars. The *Q*_m_ of biochars decreased after aging treatment, except for Cd^2+^ adsorption capacity of CCB350 in the binary metal system. This showed that the adsorption capacity had a tendency to decrease for most biochars when aged with H_2_O_2_. A similar trend was previously reported^35,55^. This might be due to the fact that aging introduced more O-containing functional groups, which could connect neighboring water molecules via strong H-bonding to form three dimensional water clusters. Thus, these clusters impeded the combination of heavy metal irons and functional groups on the surface of the biochar^56^.

### Effects of aging on the adsorption kinetics of Cd^2+^and Pb^2+^ by biochars

**Figure 2:**
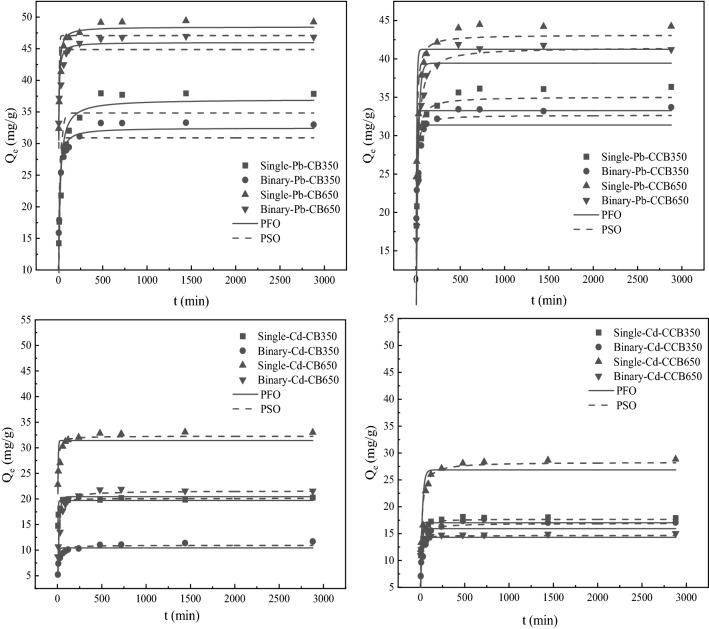
Adsorption kinetics of Pb^2+^ and Cd^2+^ on the fresh and aged biochars in the single and binary systems.

Adsorption kinetics of Pb^2+^ and Cd^2+^ on fresh and aged biochars in the systems is shown in Fig. 2. It was evident that Pb^2+^ and Cd^2+^ adsorption on biochars rose sharply in the first 0–120 min, rose at a slow level for the next 120–720 min, and reached equilibrium in 720–2280 min. The adsorption process has three stages: (1) the rapid adsorption process, (2) the steady adsorption process, and (3) the process in equilibrium. The multi-linearity of the intra-particle diffusion plot suggested that the whole adsorption process was composed of three stages (Fig. S4). In the first stage, instantaneous adsorption resulted in the sharper portion in the graph. The Pb^2+^ and Cd^2+^ transferred from the aqueous phase to the external biochars surface. In the second step, there was internal diffusion of Pb^2+^ and Cd^2+^ through the pores of biochars. It showed a decrease in the slope due to the gradual decrease in the adsorption rate. The third region was the final equilibrium stage. The whole adsorption process was controlled by a rapid first stage, a slower second stage, and a much slower third stage until reaching equilibrium. At the end of the fast phase of adsorption, the adsorption amounts of Cd^2+^ and Pb^2+^ by biochars accounted for about 84.60–97.57% of the total adsorption amounts. These results showed that the rapid stage in adsorption was more prominent in this process. Aging treatment was without vital effect on the equilibrium time of heavy metals in adsorption.

The data were fitted by the model of pseudo-first-order (PFO) and pseudo-second-order (PSO), and the relevant parameters are shown in Table S3. The coefficient of determination (*R*^2^) obtained from the pseudo-first-order models (0.5617–0.9424) was lower than that of the pseudo-second-order model (0.9050–0.9786) for all biochars. Pb^2+^ and Cd^2+^ adsorption on fresh and aged biochars was more consistent with the pseudo-second-order model, suggesting that chemisorption (ion exchange, complexation, and precipitation) was the main rate-limiting step in this adsorption process^57^. Moreover, the calculated values (*Q*_e_) of the pseudo-second-order model were closer to the values of experiment (*Q*_exp_).

To further investigate the rate limiting steps involved in the adsorption process, the intra-particle diffusion (IPD) model was adopted in a kinetics analysis. In this study, data for the Cd^2+^ and Pb^2+^ adsorption on the fresh and aged biochars in the single and binary systems within 720 min were well fitted by the IPD model (Table S4), suggesting that intra-particle diffusion played a significant role in Cd^2+^ and Pb^2+^ adsorption. The sorption rate in stage 1 (*k*_i1_) was much higher than that in other stages (*k*_i2_ and *k*_i3_), reflecting the external diffusion was the rate-determining step during sorption. Diffusion resistance was evaluated by the intercept *C* of each stage, which provided information about the thickness of the boundary layer^58^. The intercept *C* of each stage followed the order of *C*_1_ (first stage) < *C*_2_ (second stage) < *C*_3_ (third stage). It indicated that external diffusion resistance was the lowest in early stages.

### Effects of aging on the adsorption mechanisms of Pb^2+^ and Cd^2+^ by biochars

#### Cation exchange

**Figure 3:**
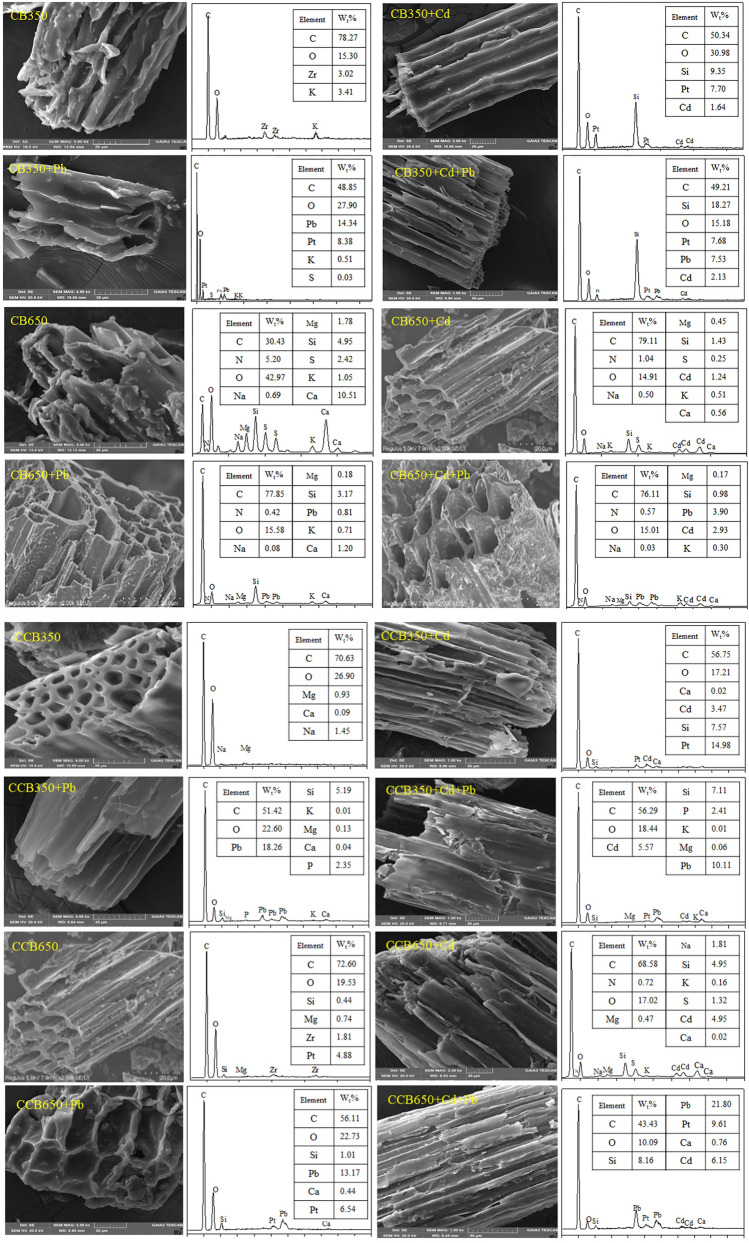
SEM images (left) and corresponding EDS spectra (right) of fresh and aged biochars produced at 350 °C and 650 °C (CB350, CB650, CCB350, and CCB650, respectively) before and after reaction with Cd^2+^ and Pb^2+^ in the single and binary systems.

**Figure 4:**
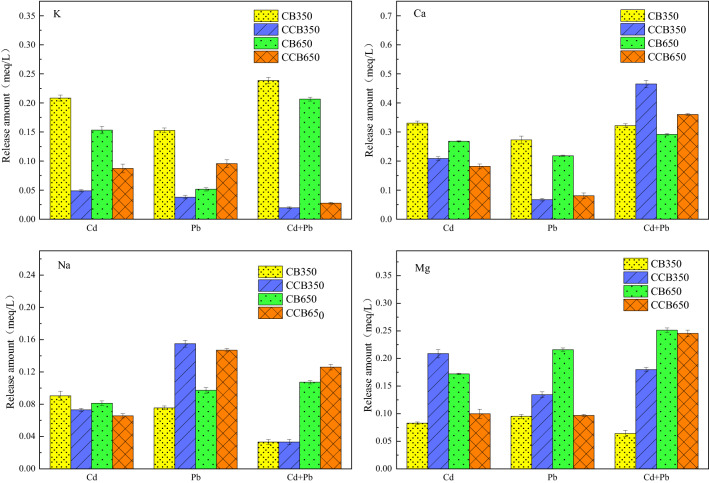
Net release amounts of K^+^, Ca^2+^, Na^+^, and Mg^2+^ from biochars before and after adsorption.

Many cations are remained on the surface of the biochar by direct electrostatic attraction, complexation or precipitation. In the process of the adsorption, these cations on the biochar are in exchange for Cd^2+^ and Pb^2+^ in solution through a cation exchanging reaction^20,39^. To illustrate this possible mechanism, the contents of cations in the solutions before and after adsorption of Pb^2+^ and Cd^2+^ by biochar (CB350, CB650, CCB350, and CCB650) were determined by SEM–EDS (Fig. 3). The percentage contents of Pb^2+^ and Cd^2+^ elements on the surface of biochars increased after adsorption, while the percentage contents of the metal elements (K^+^, Ca^2+^, Na^+^ and Mg^2+^) decreased or even lowered to the limit of detection. To further quantify the amount of the metals from the solution before and after the adsorption of Pb^2+^ and Cd^2+^ by biochar in single and binary metal systems, we calculated the net release amount of metal ions (Fig. 4). After adsorption of Pb^2+^ and Cd^2+^, the net release amounts of the elements from the single and binary metal systems were 0.0196–0.2385, 0.0806–0.4648, 0.0333–0.1550, and 0.0639–0.2454 meq/L, respectively. The net emissions of Ca^2+^ and Mg^2+^ were much greater than those of K^+^ and Na^+^, indicating a vital role in the cation exchange process. This was mainly because K^+^ and Na^+^ could only be retained on the surface of biochars by external spherical electrostatic attraction to the negatively charged position on the surface. However, Ca^2+^ and Mg^2+^ remained on biochar due to precipitation (CaCO_3_ or MgCO_3_) or complexation of inner sphere for the acid groups on the surface of biochar^59^.

In comparison with the fresh biochars, the total release amounts of K^+^, Ca^2+^, Na^+^ and Mg^2+^ of the metal systems obviously decreased after absorbing the Pb^2+^ and Cd^2+^ by aged biochars, which may be related to the leaching of soluble salt ions during the aging process^60^.

#### Complexation with oxygen-containing functional groups

**Figure 5:**
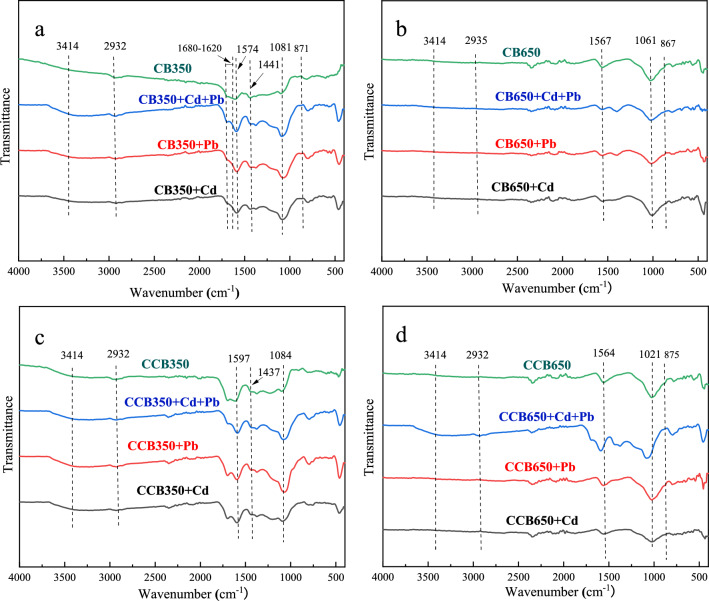
FTIR spectra of biochars before and after reaction with Cd^2+^ and Pb^2+^ in the single and binary systems (**a**) for CB350, (**b**) for CB650, (**c**) for CCB350, and (**d**) for CCB650).

The oxygenated groups (e.g., –OH, –COOH, –R–OH) distributed on surfaces of the biochar formed metal complexes with heavy metal ions, which reduced the concentration and mobility of heavy metals^61^. A lot of studies have revealed that the complexation between heavy metals and oxygenated functional groups is a significant mechanism for biochar adsorption^20,39,62^. The changes of these groups in biochar adsorption were analyzed by FTIR (Fig. 5). The dominant bands at 3414 cm^−1^, 2932 cm^−1^, 1701–1680 cm^−1^, 1564–1597 cm^−1^ and 1021–1084 cm^−1^ represented –OH, C–H of aromatic groups, –COOH, C=O/C=C, and C–O–C, respectively^23,61^. These functional groups were transformed after the adsorption. For CB650, the intensity of the stretching vibration peak of –OH did not change evidently after the adsorption of Pb^2+^ and Cd^2+^, which was due to the low content of –OH in the high-temperature biochar. However, the intensity and peak position of –OH changed after the adsorption by CCB350 and CCB650, indicating that –OH was in the process of the adsorption of these metals^28^. The intensity of aromatic C–H weakened after the heavy metal adsorption by CB350 and CCB350, while CCB650 + Pb + Cd was enhanced, indicating that the aromatic group C–H was in the adsorption of Pb^2+^ and Cd^2+^^63^. The strength of the –COOH stretching vibration peaks of CB350 + Pb, CB350 + Cd and CCB350 + Pb + Cd weakened after the adsorption of the heavy metals Pb^2+^ and Cd^2+^. The band at 1441–1437 cm^-1^ was classified to CO_3_^2−^, at 1104 cm^−1^ was because of the P–O stretching vibrations^64^, which may be due to the metallic carbonate and metallic phosphate precipitates formation, respectively. The C=O/C=C intensities of CB650 + Pb, CB650 + Cd, CB650 + Pb + Cd, CCB650 + Pb, and CCB650 + Cd were weakened. The stretching vibration peak of C–O–C showed a small shift and change. These results indicated that these groups were in the adsorption of Pb^2+^ and Cd^2+^ by biochar.

#### Precipitation with minerals

Some inorganic components of biochar (CO_3_^2−^, SO_4_^2−^, OH^−^, and PO_4_^3−^) can also be precipitated with heavy metal irons^65^. The function of these anions in the adsorption of Pb^2+^ and Cd^2+^ on biochar has been reported by Fei et al.^66^. In the present study, after adsorbing heavy metals, SEM results showed that white granular crystals were formed on the surface of biochar. Furthermore, EDS analysis believed that the white granular crystals involved Cd and Pb. Hence, the ability of mineral precipitation to adsorb heavy metals cannot be ignored.

**Figure 6:**
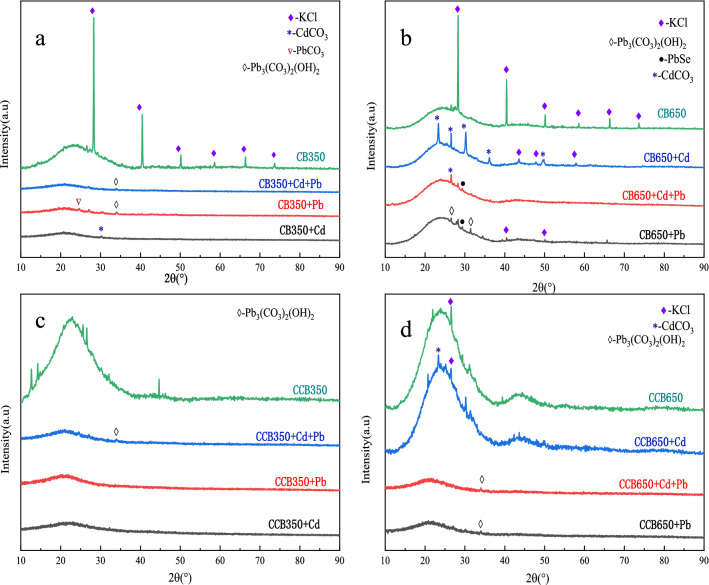
XRD patterns of biochars before and after reaction with Cd^2+^ and Pb^2+^ in the single and binary systems (**a** for CB350, **b** for CB650, **c** for CCB350, and **d** for CCB650).

To further determine their precipitate crystals, all biochars before and after the adsorption of Pb^2+^ and Cd^2+^ were scanned by XRD (Fig. 6). The XRD patterns also showed the presence of CdCO_3_, PbCO_3_, Pb_3_(CO_3_)_2_(OH)_2_, and PbSe on the surface of the biochars after adsorption. Biochar is rich in inorganic minerals. The higher the ash contents of biochar, the stronger the precipitation of heavy metals^67^. The ash contents of biochar at high temperature (CB650 and CCB650) were higher than those of low-temperature biochars (CB350 and CCB350). Similarly, the ash content of the fresh biochars (CB350 and CB650) was greater than that of the aged biochars (CCB350, CCB650). This showed that a low pyrolysis temperature and aging could reduce the precipitation of heavy metals.

#### Other potential mechanisms

Except for cation exchange, complexation, and precipitation, there are other adsorption mechanisms^68^. Except for functional groups (–OH, –COOH, –R–OH), the influence of other functional groups (γ-CH, C=C) and basic groups (C=N, –NH_2_) on the adsorption of Pb^2+^ and Cd^2+^ by biochar cannot be neglected^38^. More aromatic structures were formed in the biochar with a rising pyrolysis temperature, which could allow the donation of π-electron to interact with Pb^2+^ and Cd^2+^, resulting in an excellent concordant affinity for heavy metals^69^. Figure 5 showed that in comparison with low-temperature biochar (CB350, CCB350), the high temperature biochar (CB650, CCB650) caused more changes in aromatic C–H functional groups at 400–700 cm^-1^ after adsorption, indicating that more aromatic functional groups, such as the γ-CH of furan and the β-ring of pyridine. This was similar to previous results^70,71^.

Furthermore, biochar is able to adsorb heavy metal cations (Cd^2+^, Pb^2+^, Zn^2+^, Cu^2+^, Ni^2+^, etc.) due to its negative surface charge^72^. To elucidate the role played by electrostatic attraction under experimental conditions, the surface zeta potential values of CB350, CB650, CCB350 and CCB650 were examined at pH 5. The zeta-potential values for CB350, CB650, CCB350 and CCB650 were − 53.5 mV, − 45.5 mV, − 57.5 mV and − 56.4 mV, respectively, indicating that all biochar surfaces were negatively charged under these experimental conditions. Therefore, electrostatic attraction was expected to generate between positively charged metal cations and negatively charged functional groups.

Microporous structures and the specific surface area is important for the biochar physical absorption^72,73^. In this experiment, the SSA of high temperature and aged biochar was greater than that of low temperature and fresh biochar, so physical adsorption might play a major role in high temperature and aged biochar.

### Effects of aging on the quantitative contribution of adsorption mechanisms by biochars

**Figure 7:**
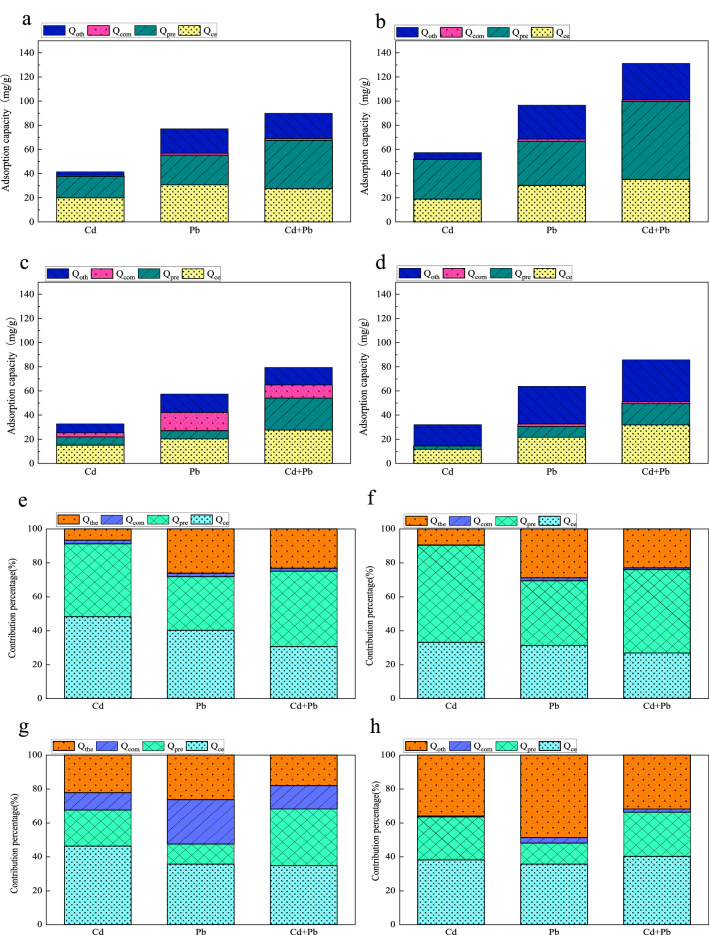
The quantitative contribution of *Q*_ce_, *Q*_com_, *Q*_pre_ and *Q*_oth_ to the adsorption of Cd^2+^ and Pb^2+^ on biochar: (**a** and **e** for CB350, **b** and **f** for CB650, **c** and **g** for CCB350, and **d** and **h** for CCB650, respectively).

The contribution of cation exchange (*Q*_ce_), precipitation with minerals (*Q*_pre_), surface complexation (*Q*_com_) and other mechanisms (*Q*_oth_) within the fresh and aged biochars to the adsorption of the metals in the systems were calculated (Fig. 7). Cation exchange was the major adsorption mechanism for the fresh low-temperature biochar (CB350) in the single system. The values of *Q*_ce_ for Cd^2+^ and Pb^2+^ reached 20.02 and 30.89 mg/g, accounting for 48.23% and 40.07% of the total adsorption capacity, respectively. The contribution of *Q*_ce_ was going down with the increase of pyrolysis temperature. This might be due to the fact that mineral crystallization inhibits the release of cations at high pyrolysis temperatures^74^. The main adsorption mechanism of CB650 in the single system changed from cation exchange to mineral precipitation. The *Q*_pre_ of Cd^2+^ and Pb^2+^ were 32.84 and 36.76 mg/g, corresponding to 57.19% and 38.04% of the adsorption contribution, respectively. Under competitive conditions, the *Q*_ce_/*Q*_t_ values were at a lower level, compared to those in single metal systems. Precipitation with minerals formed the biggest fraction for CB350 and CB650 in the binary metal system, which contributed 44.36% and 49.20%, respectively.

After aging, the values of *Q*_com_ and *Q*_com_/*Q*_t_ for CCB350 and CCB650 increased to varying degrees under both single and binary metal systems, and this phenomenon was more obvious for low-temperature biochar. For CB350, the *Q*_com_/*Q*_t_ values of Cd^2+^ and Pb^2+^ were 2.12% and 0.28%, respectively. However, after aging, the *Q*_com_/*Q*_t_ values of Cd^2+^ and Pb^2+^ showed 10.20% and 0.53%, respectively, which increased by 8.08% and 0.25%, respectively. Further, the *Q*_pre_ and *Q*_pre_/*Q*_t_ values of CCB350 and CCB650 decreased significantly compared with fresh biochars in all systems, and the *Q*_oth_/*Q*_t_ values of CCB650 increased significantly after aging.

The above results showed that cation exchange (*Q*_ce_) belonged to the main mechanism for CB350 and CCB350 (low temperature biochar) in a single-metal system, with the *Q*_ce_/*Q*_t_ values ranging from 35.63% to 48.23%. Precipitation with minerals (*Q*_pre_) and other mechanisms (*Q*_oth_) were foremost for CB650 and CCB650 in the single-metal system, respectively. The competitive interaction between Cd^2+^ and Pb^2+^ increased the relative contribution of mineral precipitation. Aging resulting in the contribution of surface complexation to Cd^2+^ and Pb^2+^ adsorption in all biochars, especially in low temperature biochars but reduced the contribution of mineral precipitation to adsorption. Notably, the contribution of other mechanisms to the adsorption increased significantly after the aging of the high-temperature biochar.

## Conclusions

The aging of corn straw biochar significantly changed its physicochemical characteristics, affecting the competitive adsorption behavior and mechanism of Cd^2+^ and Pb^2+^. In the single-metal system, cation exchange was the key adsorption mechanism for biochar (35.63–48.23%) at a low temperature. However, mineral precipitation dominated Cd^2+^ and Pb^2+^ adsorption (38.04–57.19%) on fresh high-temperature biochars. Competitive interactions between Cd^2+^ and Pb^2+^ increased the relative contribution of mineral precipitation, but the contribution of cation exchange mechanism was reduced. Biochar aging increased the contribution of surface complexation to the adsorption of Cd^2+^ and Pb^2+^ on biochars, especially in low-temperature biochars, but weakened the contribution of mineral precipitation to the adsorption. Further, the contribution of other adsorption mechanisms was significantly enhanced for high-temperature aged biochars. Therefore, the results in this study are expected to provide a theoretical basis for the long term application of biochar in the remediation of heavy metal compound polluted soil.

## Supplementary Information

Supplementary Information.

## Data Availability

The data used to support the findings of this study are included within the article and the supplementary information files.

## References

[1] Bashir S, Zhu J, Fu QL, Hu HQ (2018). Cadmium mobility, uptake and anti-oxidative response of water spinach (Ipomoea aquatic) under rice straw biochar, zeolite and rock phosphate as amendments. Chemosphere.

[2] Fajardo C, Costa G, Nande M, Martín C, Martín M, Sánchez-Fortún S (2019). Heavy metals immobilization capability of two iron-based nanoparticles (nZVI and Fe_3_O_4_): Soil and freshwater bioassays to assess ecotoxicological impact. Sci. Total. Environ..

[3] Sellaoui L, Soetaredjo FE, Ismadji S, Bonilla-Petriciolet A, Belver C, Bedia J, Lamine AB, Erto A (2018). Insights on the statistical physics modeling of the adsorption of Cd^2+^ and Pb^2+^ ions on bentonite-chitosan composite in single and binary systems. Chem. Eng. J..

[4] Yan YB, Li Q, Sun XY, Ren ZY, He F, Wang YL, Wang LJ (2015). Recycling flue gas desulphurization (FGD) gypsum for removal of Pb(II) and Cd(II) from waste water. J. Colloid Interface Sci..

[5] Lian C, Zhou SL, Shi YX, Wang CH (2017). Heavy metals in food crops, soil, and water in the Lihe River Watershed of the Taihu Region and their potential health risks when ingested. Sci. Total. Environ..

[6] Jeon EK, Jung JM, Kim WS, Ko SH, Beak K (2015). In situ electrokinetic remediation of As-, Cu-, and Pb-contaminated paddy soil using hexagonal electrode configuration: A full scale study. Environ. Sci. Pollut. Res..

[7] Chen D, Liu XY, Bian RJ, Cheng K, Zhang XH, Zheng JF, Joseph S, Crowley D, Pan GX, Li LQ (2018). Effects of biochar on availability and plant uptake of heavy metals: A meta-analysis. J. Environ. Manag..

[8] Cheng S, Chen T, Xu WB, Huang J, Jiang SJ, Yan B (2020). Application research of biochar for the remediation of soil heavy metals contamination: A review. Molecules.

[9] El-Naggar A, Lee SS, Rinklebe J, Farooq M, Hocheol S, Sarmah AK, Zimmerman AR, Ahmad M, Shaheen SM, Ok YS (2019). Biochar application to low fertility soils: A review of current status, and future prospects. Geoderma.

[10] Wang L, Ok YS, Tsang DCW, Alessi DS, Rinklebe J, Wang H, Mašek O, Hou R, O’Connor D, Hou D (2020). New trends in biochar pyrolysis and modification strategies: Feedstock, pyrolysis conditions, sustainability concerns and implications for soil amendment. Soil Use Manag..

[11] Wang LW, O’Connor D, Rinklebe J, Ok YS, Tsang DCW, Shen ZT, Hou DY (2020). Biochar aging: Mechanisms, physicochemical changes, assessment, and implications for field applications. Environ. Sci. Technol..

[12] Gholizadeh M, Hu X (2021). Removal of heavy metals from soil with biochar composite: A critical review of the mechanism. J. Environ. Chem. Eng..

[13] Ni BJ, Huang QS, Wang C, Ni TY, Sun J, Wei W (2019). Competitive adsorption of heavy metals in aqueous solution onto biochar derived from anaerobically digested sludge. Chemosphere.

[14] Abdin Y, Usman A, Ok YS, Tsang YF, Al-Wabel M (2019). Competitive sorption and availability of coexisting heavy metals in mining-contaminated soil: Contrasting effects of mesquite and fishbone biochars. Environ. Res..

[15] Han L, Qian LB, Liu RQ, Chen MF, Yan JC, Hu QH (2017). Lead adsorption by biochar under the elevated competition of cadmium and aluminum. Sci. Rep..

[16] Deng YY, Huang S, Laird DA, Wang XG, Meng ZW (2019). Adsorption behaviour and mechanisms of cadmium and nickel on rice straw biochars in single- and binary-metal systems. Chemosphere.

[17] Fan SS, Li H, Wang Y, Wang Z, Tang J, Tang J, Li XD (2017). Cadmium removal from aqueous solution by biochar obtained by co-pyrolysis of sewage sludge with tea waste. Res. Chem. Intermed..

[18] Sun JK, Fei L, Liu ZQ, Zhu LY, Song ZG (2014). Biochars derived from various crop straws: Characterization and Cd(II) removal potential. Ecotoxicol. Environ. Saf..

[19] Chang RH, Sohi SP, Jing FQ, Liu YY, Chen JW (2019). A comparative study on biochar properties and Cd adsorption behavior under effects of ageing processes of leaching, acidification and oxidation. Environ. Pollut..

[20] Lu HL, Zhang WH, Yang YX, Huang XF, Wang SZ, Qiu RL (2012). Relative distribution of Pb^2+^ sorption mechanisms by sludge-derived biochar. Water Res..

[21] Liu L, Fan SS (2018). Removal of cadmium in aqueous solution using wheat straw biochar: Effect of minerals and mechanism. Environ. Sci. Pollut. Res..

[22] Wang ZY, Liu GC, Zheng H, Li FM, Ngo HH, Guo WS, Liu C, Chen L, Xing BS (2015). Investigating the mechanisms of biochar's removal of lead from solution. Bioresour. Technol..

[23] Tan LS, Ma ZH, Yang KQ, Cui QL, Wang K, Wang TT, Wu GL, Zheng JY (2020). Effect of three artificial aging techniques on physicochemical properties and Pb adsorption capacities of different biochars. Sci. Total. Environ..

[24] Fu Q, Zhao H, Li TX, Hou RJ, Liu D, Ji Y, Zhou ZQ, Yang LY (2019). Effects of biochar addition on soil hydraulic properties before and after freezing-thawing. CATENA.

[25] Meng ZW, Huang S, Xu T, Deng YY, Lin ZB, Wang XG (2020). Transport and transformation of Cd between biochar and soil under combined dry-wet and freeze-thaw aging. Environ. Pollut..

[26] Quan GX, Fan QY, Cui LQ, Zimmerman AR, Wang HL, Zhu ZY, Gao B, Wu LM, Yan JL (2020). Simulated photocatalytic aging of biochar in soil ecosystem: Insight into organic carbon release, surface physicochemical properties and cadmium sorption. Environ. Res..

[27] Hua Y, Zheng XB, Xue LH, Han LF, He SY, Mishra T, Feng YF, Yang LZ, Xing BS (2020). Microbial aging of hydrochar as a way to increase cadmium ion adsorption capacity: Process and mechanism. Bioresour. Technol..

[28] Huff MD, Lee JW (2016). Biochar-surface oxygenation with hydrogen peroxide. J. Environ. Manag..

[29] Li HY, Ye XX, Geng ZG, Zhou HJ, Guo XS, Zhang YX, Zhao HJ, Wang GZ (2016). The influence of biochar type on long-term stabilization for Cd and Cu in contaminated paddy soils. J. Hazard. Mater..

[30] Mia S, Dijkstra FA, Singh B (2017). Aging induced changes in biochar’s functionality and adsorption behavior for phosphate and ammonium. Environ. Sci. Technol..

[31] Ren XH, Sun HW, Wang F, Cao FM (2016). The changes in biochar properties and sorption capacities after being cultured with wheat for 3 months. Chemosphere.

[32] Shi KS, Xie Y, Qiu YP (2015). Natural oxidation of a temperature series of biochars: Opposite effect on the sorption of aromatic cationic herbicides. Ecotoxicol. Environ. Saf..

[33] Hale SE, Hanley K, Lehmann J, Zimmerman AR, Cornelissen G (2011). Effects of chemical, biological, and physical aging as well as soil addition on the sorption of pyrene to activated carbon and biochar. Environ. Sci. Technol..

[34] Li HX, Lu XQ, Xu Y, Liu HT (2019). How close is artificial biochar aging to natural biochar aging in fields? A meta-analysis. Geoderma.

[35] Nie TT, Hao PL, Zhao ZD, Zhou WJ, Zhu LJ (2019). Effect of oxidation-induced aging on the adsorption and co-adsorption of tetracycline and Cu^2+^ onto biochar. Sci. Total Environ..

[36] Cao XD, Ma L, Gao B, Harris W (2009). Dairy-manure derived biochar effectively sorbs lead and atrazine. Environ. Sci. Technol..

[37] Deng YY, Huang S, Laird DA, Wang XG, Dong CQ (2018). Quantitative mechanisms of cadmium adsorption on rice straw-and swine manure-derived biochars. Environ. Sci. Pollut. Res..

[38] Huang F, Gao LY, Wu RR, Wang H, Xiao RB (2020). Qualitative and quantitative characterization of adsorption mechanisms for Cd^2+^ by silicon-rich biochar. Sci. Total. Environ..

[39] Cui XQ, Fang SY, Yao YQ, Li TQ, Yang XE, He ZL (2016). Potential mechanisms of cadmium removal from aqueous solution by Canna indica derived biochar. Sci. Total. Environ..

[40] Ho SH, Chen YD, Yang ZK, Chen MF, Yan JC, Hu QH (2017). High-efficiency removal of lead from wastewater by biochar derived from anaerobic digestion sludge. Bioresour. Technol..

[41] Deng YY, Huang S, Dong CQ, Meng ZW, Wang XG (2020). Competitive adsorption behaviour and mechanisms of cadmium, nickel and ammonium from aqueous solution by fresh and ageing rice straw biochars. Bioresour. Technol..

[42] Cao XD, Harris W (2010). Properties of dairy-manure-derived biochar pertinent to its potential use in remediation. Bioresour. Technol..

[43] Shinogi Y, Kanri Y (2003). Pyrolysis of plant, animal and human waste: Physical and chemical characterization of the pyrolytic products. Bioresour. Technol..

[44] Zhao YQ, Huang L, Chen YC (2017). Biochars derived from giant reed (*Arundo donax* L.) with different treatment: characterization and ammonium adsorption potential. Environ. Sci. Pollut. Res..

[45] Qian LB, Chen MF, Chen BL (2015). Competitive adsorption of cadmium and aluminum onto fresh and oxidized biochars during aging processes. J. Soil. Sediment..

[46] Mia S, Dijkstra FA, Singh B (2017). Long-term aging of biochar: A molecular understanding with agricultural and environmental implications. Adv. Agron..

[47] Yang K, Wang XL, Cheng HF, Tao S (2021). Effect of aging on stabilization of Cd and Ni by biochars and enzyme activities in a historically contaminated alkaline agricultural soil simulated with wetedry and freezeethaw cycling. Environ. Pollut..

[48] Sanford JR, Larson RA, Runge T (2019). Nitrate sorption to biochar following chemical oxidation. Sci. Total Environ..

[49] Jing FQ, Sohi SP, Liu YY, Chen JW (2018). Insight into mechanism of aged biochar for adsorption of PAEs: Reciprocal effects of ageing and coexisting Cd^2+^. Environ. Pollut..

[50] Park JH, Choppala G, Lee SJ, Bolan N, Chung JW, Edraki M (2013). Comparative sorption of Pb and Cd by biochars and its implication for metal immobilization in soils. Water Air Soil Pollut..

[51] Jeppu GP, Clement TP (2012). A modified Langmuir–Freundlich isotherm model for simulating pH-dependent adsorption effects. J. Contam. Hydrol..

[52] Liu YY, Wang L, Wang XY, Jing FQ, Chang RH, Chen JW (2020). Oxidative ageing of biochar and hydrochar alleviating competitive sorption of Cd(II) and Cu(II). Sci. Total. Environ..

[53] Park JH, Ok YS, Kim SH, Cho JS, Heo JS, Delaune RD, Seo DC (2016). Competitive adsorption of heavy metals onto sesame straw biochar in aqueous solutions. Chemosphere.

[54] Ding ZH, Xin H, Wan YS, Wang SS, Gao B (2016). Removal of lead, copper, cadmium, zinc, and nickel from aqueous solutions by alkali-modified biochar: Batch and column tests. J. Ind. Eng. Chem..

[55] Wu WD, Li JH, Lan T, Müller K, Niazi NK, Chen X, Xu S, Zheng LR, Chu YC, Li JW, Yuan GD, Wang HL (2017). Unraveling sorption of lead in aqueous solutions by chemically modified biochar derived from coconut fiber: A microscopic and spectroscopic investigation. Sci. Total. Environ..

[56] Ghaffar A, Abbas G (2016). Adsorption of phthalic acid esters (PAEs) on chemically aged biochars. Green Process. Synth..

[57] Ahmad Z, Gao B, Mosa A, Yu HW, Yin XQ, Bashir A, Ghoveisi H, Wang SS (2018). Removal of Cu(II), Cd(II) and Pb(II) ions from aqueous solutions by biochars derived from potassium-rich biomass. J. Clean. Prod..

[58] Tan X, Liu Y, Gu Y, Zeng G, Hu X, Wang X, Guo Y, Zeng X, Sun Z (2015). Biochar amendment to lead-contaminated soil: Effects on fluorescein diacetate hydrolytic activity and phytotoxicity to rice. Environ. Toxicol. Chem..

[59] Zhang F, Wang X, Yin DX, Peng B, Tan CY, Liu YG, Tan XF, Wu SX (2015). Efficiency and mechanisms of Cd removal from aqueous solution by biochar derived from water hyacinth (*Eichornia crassipes*). J. Environ. Manag..

[60] Bakshi S, Aller DM, Laird DA, Chintala R (2016). Comparison of the physical and chemical properties of laboratory and field-aged biochars. J. Environ. Qual..

[61] Fan QY, Sun JX, Chu L, Cui LQ, Quan GX, Yan JL, Hussain Q, Iqbal M (2018). Effects of chemical oxidation on surface oxygen-containing functional groups and adsorption behavior of biochar. Chemosphere.

[62] Huang F, Wen XH, Cai YX, Cai KZ (2018). Silicon-mediated enhancement of heavy metal tolerance in rice at different growth stages. Int. J. Environ. Res. Public Health.

[63] Lawrinenko M, Laird DA, Johnson RL, Jing D (2016). Accelerated aging of biochars: Impact on anion exchange capacity. Carbon.

[64] Qian LB, Chen BL (2013). Dual role of biochars as adsorbents for aluminum: The effects of oxygen-containing organic components and the scattering of silicate particles. Environ. Sci. Technol..

[65] Gao RL, Xiang L, Hu HQ, Fu QL, Zhu J, Liu YH, Huang GY (2020). High-efficiency removal capacities and quantitative sorption mechanisms of Pb by oxidized rape straw biochars. Sci. Total. Environ..

[66] Fei H, Gao LY, Deng JH, Chen SH, Cai KZ (2018). Quantitative contribution of Cd^2+^ adsorption mechanisms by chicken-manure-derived biochars. Environ. Sci. Pollut. Res..

[67] Zhang C, Shan BQ, Tang WZ, Zhu YY (2017). Comparison of cadmium and lead sorption by Phyllostachys Pubescens biochar produced under a low-oxygen pyrolysis atmosphere. Bioresour. Technol..

[68] Mahdi Z, Yu QJ, Hanandeh AE (2018). Investigation of the kinetics and mechanisms of nickel and copper ions adsorption from aqueous solutions by date seed derived biochar. J. Environ. Chem. Eng..

[69] Yu WC, Lian F, Cui GN, Liu ZQ (2018). N-doping effectively enhances the adsorption capacity of biochar for heavy metal ions from aqueous solution. Chemosphere.

[70] Gao LY, Deng JH, Huang GF, Li K, Cai KZ, Liu Y, Huang F (2018). Relative distribution of Cd^2+^ adsorption mechanisms on biochars derived from rice straw and sewage sludge. Bioresour. Technol..

[71] Uchimiya M, Lima IM, Klasson KT, Chang SC, Wartelle LH, Rodgers JE (2010). Immobilization of heavy metal ions (CuII, CdII, NiII, and PbII) by broiler litter-derived biochars in water and soil. J. Agric. Food Chem..

[72] Tran HN, You SJ, Hosseini-Bandegharaei A, Chao HP (2017). Mistakes and inconsistencies regarding adsorption of contaminants from aqueous solutions: A critical review. Water Res..

[73] Shen ZT, Zhang YY, Jin F, McMillan O, Al-Tabbaa A (2017). Qualitative and quantitative characterization of adsorption mechanisms of lead on four biochars. Sci. Total. Environ..

[74] Wang RZ, Huang DL, Liu YG, Zhang C, Lai C, Zeng GM, Cheng M, Gong XM, Wan J, Luo H (2018). Investigating the adsorption behavior and the relative distribution of Cd^2+^ sorption mechanisms on biochars by different feedstock. Bioresour. Technol..

