# Supplementary material for: Effect of oxidative aging of biochar on relative distribution of competitive adsorption mechanism of Cd2+ and Pb2+
Source: Sci Rep. 2022 Jul 4;12:11308. doi: 10.1038/s41598-022-15494-y (PMC9252994; doi:10.1038/s41598-022-15494-y)
Supplement: Supplementary file 1 — Supplementary Information. [file 41598_2022_15494_MOESM1_ESM.docx]

# Supplementary Material

**Effect of oxidative aging of biochar on relative distribution of competitive adsorption mechanism of Cd^2+^ and Pb^2+^**

Zhe Wang^a,1^, Chengxin Geng^a,1^, Yuan Bian^a^, Guangyu Zhang^a^, Chunli Zheng^a,b*^,Chunjiang An^c^

*a School of Energy and Environment, Inner Mongolia University of Science and Technology, Baotou 014010, China*

*b* *Inner Mongolia Engineering Research Center of Evaluation and Restoration in the Mining Ecological Environment, Inner Mongolia University of Science and Technology, Baotou 014010, China*

*c Department of Building, Civil and Environmental Engineering, Concordia University, Montreal, QC H3G 1M8, Canada*

*Corresponding author: zhengchunli1979@163.com

^1^ Co-first author

Number of pages: 12

Number of tables: 4

Number of figures: 4

**Materials and Methods**

*Chemical regents*

Cd(NO_3_)_2_ 4H_2_O、Pb(NO_3_)_2_、NaNO_3_、H_2_O_2_ (30%)、NaOH、HNO_3_、HCl were obtained from Tianjin Chemical Reagent No. 3 Factory, Tianjin, China. The experiment used reagents at the analytical level. Specifically, all solutions are configured as deionized water (>18.3 MΩ cm).

*Preparation of biochars*

Corn straw samples were collected from Baotou, Inner Mongolia, China. The raw materials were first washed three times with deionized water to remove dust, and then dried at 80 ℃ for 48 h, followed by grinding in a stainless grinding machine and sieving to < 2.0 mm particles. The sieved biomass particles were compacted into a ceramic pot, covered with a lid and sealed with tin foil to promote an oxygen-limited environment during heating. A muffle furnace (SK16BYL, Nanjing Boyun Tong Instrument Technology Company, Nanjing, China) was applied to produce biochars under N_2_ atmosphere at a flow rate of 50 mL/min. The pyrolysis temperature was increased to 350 ℃ and 650 ℃ with a heating rate 5 ℃/min, and maintained for 3 h. After natural cooling, the biochar products were grounded until the whole sample passed through a 100 mesh sieve. The biochars prepared at 350 ℃ and 650 ℃ were stored in a desiccator and recorded as CB350 and CB650, respectively.

*Characterization of biochars*

Firstly，the ash (burned for 4 h in an open crucible at 750 °C) and pH (1/20 water extraction) of the biochars were calculated. The surface charge properties of biochars were evaluated by zeta potential measurements, which were conducted at different equilibrium pH using an electroacoustic spectrometer (ZEN3600 Zetasizer, UK). The elemental composition of biochar was constructed by elemental analysis (Flash Smart, Thermo Fisher, Italy). Element O was calculated on the basis of the C, H, N, and S and ash determinations. Fourier transform infrared spectroscopy (FTIR) measured surface functional groups of biochar samples (Nicolet iS10, Thermal Fisher Scientific, Massachusetts, USA). The X-ray photoelectron spectroscopy (XPS) (AXIS Ultra DLD, Kratos, England) was chosen for quantitative analysis of biochar functional group. The surface morphology and elemental analysis were carried by scanning electron microscopy and energy dispersive spectroscopy (SEM-EDS) (Nova NanoSEM 450, FEI, USA). Crystal minerals of biochar (D8 Advance, Bruker, Germany) were analyzed by X-ray diffractometry (XRD) at 2θ with a sampling range from 10° to 90° at a scanning speed of 4º/min. According to the Brunauer-Emmett-Teller (BET) method of Gemini 2360 Micromeritics, the specific surface area (SSA) of each biochar was determined by N_2_ adsorption isotherm at 77 K.

*The models of adsorption isotherm and kinetic*

The adsorption isotherms were simulated using Langmuir (Eq. (1)), Freundlich (Eq. (2)) and Langmuir-Freundlich (Eq. (3)) isotherm models and the adsorption kinetics were fitted by Lagergren’s pseudo-first-order (PFO, Eq. (4)), pseudo-second-order (PSO, Eq. (5)) and intra-particle diffusion (IPD, Eq. (6)) kinetic equations:

Langmuir model:$Q_{\text{e}}=\frac{Q_{m}K_{L}C_{e}}{1+K_{L}C_{e}}$ （1）

Freundlich model:$\text{Q}_{\text{e}}\text{=}\text{K}_{\text{F}}{\text{C}_{\text{e}}}^{\frac{\text{1}}{\text{n}}}$ （2）

Langmuir-Freundlich model:*$Q_{e}=\frac{Q_{m}K_{LF}C_{e}^{1/n}}{1+K_{LF}C_{e}^{1/n}}$* （3）

Pseudo−first−order kinetic model:${\text{ }\text{Q}}_{\text{t}}\text{=}\text{Q}_{\text{e}}\left( \text{1-}\text{e}^{\text{-}\text{k}_{\text{1}}\text{t}} \right)$ （4）

Pseudo−second−order kinetic model:$Q_{\text{t}}=\frac{k_{2}Q_{e}^{2}t}{1+k_{2}Q_{e}t}$ （5）

Intra−particle diffusion kinetic model:$Q_{\text{t}}=k_{i}t^{0.5}+C$ （6）where *Q*_e_ and *Q*_t_ (mg/g) are the adsorption amount at equilibrium and time *t*, respectively. *Q*_m_ (mg/g) is the maximum adsorption capacity. *C*_e_ (mg/L) is the equilibrium concentration of Cd^2+^ or Pb^2+^. *K*_L_ (L/mg) and *K*_F_ (L/g) are the adsorption parameters of Langmuir and Freundlich model, respectively. *n* is an indicator of heterogeneity. *K*_LF_ is the Langmuir-Freundlich constants. *k*_1_ (l/min) and *k*_2_ (g/mg·min) are the pseudo-first-order and pseudo-second-order adsorption rate constants, respectively. *k*_i_ [mg/(g·min^0.5^)] is the rate constant of intra-particle diffusion. C (mg/g) is the intercept.

**Results and Discussion**

**Table S1** Physicochemical properties of fresh and aged biochars.

| Biochars | Ash  (%) | SSA  (m^2^/g) | pH | Elemental composition (% mass) | | | | | Atomic ratio | | |
| --- | --- | --- | --- | --- | --- | --- | --- | --- | --- | --- | --- |
|  |  |  |  | C | H | O | N | S | H/C | O/C | (O+N)/C |
| CB350 | 19.31 | 1.57 | 9.60 | 60.93 | 3.47 | 14.22 | 1.86 | 0.20 | 0.057 | 0.233 | 0.264 |
| CCB350 | 10.57 | 2.78 | 4.35 | 58.34 | 3.88 | 25.31 | 1.89 | - | 0.067 | 0.434 | 0.466 |
| CB650 | 25.17 | 203.42 | 10.85 | 59.98 | 1.31 | 11.88 | 1.41 | 0.25 | 0.022 | 0.198 | 0.222 |
| CCB650 | 14.99 | 300.69 | 9.83 | 64.37 | 1.16 | 17.72 | 1.45 | 0.31 | 0.018 | 0.275 | 0.298 |

**Table S2** Isotherm parameters of Pb^2+^ and Cd^2+^ on fresh and aged biochars in the single and binary systems.

| Biochars | System | *Q*_e_  (mg/g) | Langmuir | | | Freundlich | | | Langmuir-Freundlich | | | |
| --- | --- | --- | --- | --- | --- | --- | --- | --- | --- | --- | --- | --- |
|  |  |  | *Q*_m_  (mg/g) | *K*_L_  (L/mg) | *R*^2^ | *K*_F_  (mg/g) | *n* | *R*^2^ | *Q*_m_  (mg/g) | *K*LF | n | *R*^2^ |
| CB350 | Cd(Single) | 34.18 | 35.66 | 0.290 | 0.9490 | 11.679 | 4.008 | 0.7926 | 42.04 | 0.111 | 0.649 | 0.9240 |
|  | Pb(Single) | 81.44 | 84.91 | 0.502 | 0.9514 | 26.202 | 3.029 | 0.8867 | 110.66 | 0.293 | 1.267 | 0.7616 |
|  | Cd(double) | 19.52 | 17.83 | 0.094 | 0.9695 | 4.361 | 3.356 | 0.9121 | 39.52 | 0.088 | 1.999 | 0.6967 |
|  | Pb(double) | 44.13 | 48.87 | 0.215 | 0.9506 | 13.644 | 3.602 | 0.7515 | 44.13 | 0.173 | 0.638 | 0.8458 |
| CCB350 | Cd(Single) | 28.08 | 33.25 | 0.038 | 0.9835 | 3.719 | 2.361 | 0.9352 | 30.44 | 0.019 | 0.774 | 0.9988 |
|  | Pb(Single) | 59.09 | 58.56 | 0.017 | 0.9777 | 9.476 | 2.549 | 0.8371 | 52.01 | 0.046 | 0.698 | 0.9881 |
|  | Cd(double) | 19.10 | 18.63 | 0.077 | 0.9389 | 3.866 | 3.12 | 0.9893 | 37.54 | 0.095 | 2.136 | 0.9972 |
|  | Pb(double) | 44.39 | 46.42 | 0.035 | 0.9732 | 11.892 | 3.352 | 0.8455 | 50.20 | 0.180 | 0.979 | 0.9955 |
| CB650 | Cd(Single) | 56.18 | 65.43 | 0.091 | 0.9822 | 10.232 | 2.485 | 0.8187 | 60.61 | 0.112 | 0.951 | 0.9528 |
|  | Pb(Single) | 96.59 | 149.98 | 0.261 | 0.9647 | 29.301 | 1.591 | 0.9527 | 117.67 | 0.415 | 0.969 | 0.8361 |
|  | Cd(double) | 23.13 | 24.79 | 0.299 | 0.9791 | 7.414 | 3.825 | 0.7968 | 24.86 | 0.128 | 0.743 | 0.9564 |
|  | Pb(double) | 92.24 | 111.11 | 0.292 | 0.9227 | 24.374 | 2.022 | 0.8998 | 103.40 | 0.283 | 1.053 | 0.9084 |
| CCB650 | Cd(Single) | 26.53 | 29.69 | 0.107 | 0.9639 | 7.191 | 3.419 | 0.8448 | 27.76 | 0.062 | 0.765 | 0.9640 |
|  | Pb(Single) | 59.09 | 67.33 | 0.027 | 0.9621 | 10.500 | 2.508 | 0.9710 | 59.09 | 0.026 | 2.376 | 0.9673 |
|  | Cd(double) | 17.58 | 23.56 | 0.017 | 0.9456 | 1.700 | 2.173 | 0.9309 | 19.58 | 0.021 | 0.958 | 0.9785 |
|  | Pb(double) | 50.38 | 54.99 | 0.032 | 0.9473 | 11.097 | 2.862 | 0.8059 | 48.77 | 0.053 | 0.568 | 0.9735 |

**Table S3** Kinetic parameters of Pb^2+^ and Cd^2+^ on fresh and aged biochars in the single and binary systems.

| Biochars | System | *Q*_exp_  (mg/g) | Pseudo first order (PFO) | | | Pseudo second order (PSO) | | |
| --- | --- | --- | --- | --- | --- | --- | --- | --- |
|  |  |  | *Q*_e_  (mg/g) | *k*_1_  (1/min) | *R*^2^ | *Q*_e_  (mg/g) | *k*_2_  [g/(mg·min)] | *R*^2^ |
| CB350 | Cd(Single) | 20.28 | 19.70 | 0.579 | 0.8178 | 20.10 | 0.532 | 0.9588 |
|  | Pb(Single) | 37.88 | 34.84 | 0.110 | 0.7706 | 37.01 | 0.074 | 0.9276 |
|  | Cd(double) | 11.69 | 10.44 | 0.282 | 0.7940 | 10.95 | 0.169 | 0.9373 |
|  | Pb(double) | 32.99 | 30.91 | 0.226 | 0.8199 | 32.48 | 0.140 | 0.9565 |
| CCB350 | Cd(Single) | 17.91 | 17.04 | 0.364 | 0.8178 | 17.68 | 0.245 | 0.9143 |
|  | Pb(Single) | 36.36 | 33.27 | 0.247 | 0.7036 | 35.06 | 0.146 | 0.9050 |
|  | Cd(double) | 17.08 | 15.93 | 0.173 | 0.7159 | 16.93 | 0.103 | 0.9119 |
|  | Pb(double) | 33.70 | 31.39 | 0.354 | 0.7171 | 32.67 | 0.229 | 0.9123 |
| CB650 | Cd(Single) | 33.01 | 31.42 | 0.510 | 0.6825 | 32.28 | 0.401 | 0.9264 |
|  | Pb(Single) | 49.23 | 47.07 | 0.448 | 0.7658 | 48.44 | 0.342 | 0.9496 |
|  | Cd(double) | 21.57 | 20.45 | 0.135 | 0.8234 | 21.59 | 0.091 | 0.9537 |
|  | Pb(double) | 46.84 | 44.86 | 0.543 | 0.6975 | 45.99 | 0.447 | 0.9116 |
| CCB650 | Cd(Single) | 28.85 | 26.86 | 0.118 | 0.9424 | 28.30 | 0.084 | 0.9786 |
|  | Pb(Single) | 44.25 | 41.25 | 0.294 | 0.7204 | 43.13 | 0.185 | 0.9164 |
|  | Cd(double) | 15.02 | 14.31 | 0.581 | 0.5617 | 14.64 | 0.503 | 0.9133 |
|  | Pb(double) | 41.23 | 39.43 | 0.104 | 0.8334 | 41.52 | 0.076 | 0.9430 |

**Table S4** The parameters of intra-particle diffusion model for Pb^2+^ and Cd^2+^ on fresh and aged biochars in the single and binary systems.

| Biochars | System | Intra-particle diffusion (IPD) | | | | | | | | |
| --- | --- | --- | --- | --- | --- | --- | --- | --- | --- | --- |
|  |  | Stage 1 | | | Stage 2 | | | Stage 3 | | |
|  |  | *k_i1_*  [mg/(g· min^0.5^)] | *C*_1_ | *R*_1_2 | *k_i2_*  [mg/(g· min^0.5^)] | *C*_2_ | *R*_2_2 | *k_i3_*  [mg/(g· min^0.5^)] | *C*_3_ | *R*_3_2 |
| CB350 | Cd(Single) | 0.621 | 14.33 | 0.8484 | -0.003 | 19.95 | 0.6012 | 0.004 | 20.09 | 0.0779 |
|  | Pb(Single) | 2.217 | 10.02 | 0.9722 | 0.543 | 25.95 | 0.9947 | 0.005 | 37.65 | 0.3787 |
|  | Cd(double) | 0.564 | 4.90 | 0.8664 | 0.088 | 9.08 | 0.9607 | 0.022 | 10.05 | 0.9931 |
|  | Pb(double) | 1.883 | 12.63 | 0.9265 | 0.349 | 25.64 | 0.9987 | -0.010 | 33.56 | 0.6767 |
| CCB350 | Cd(Single) | 0.726 | 9.73 | 0.9821 | 0.032 | 17.22 | 0.9901 | -0.003 | 18.07 | 0.2277 |
|  | Pb(Single) | 1.851 | 14.55 | 0.9906 | 0.261 | 29.90 | 0.9995 | 0.009 | 35.84 | 0.6570 |
|  | Cd(double) | 0.924 | 5.82 | 0.9664 | 0.127 | 14.41 | 0.9662 | -0.023 | 18.20 | 0.6297 |
|  | Pb(double) | 1.494 | 16.97 | 0.9674 | 0.175 | 29.57 | 0.9936 | 0.012 | 32.97 | 0.4073 |
| CB650 | Cd(Single) | 1.116 | 21.09 | 0.9629 | 0.117 | 30.26 | 0.9992 | 0.010 | 32.55 | 0.4839 |
|  | Pb(Single) | 1.934 | 29.62 | 0.9537 | 0.223 | 44.20 | 0.9920 | -0.005 | 49.31 | 0.0043 |
|  | Cd(double) | 1.437 | 5.84 | 0.9872 | 0.180 | 17.85 | 0.9999 | -0.012 | 22.15 | 0.6848 |
|  | Pb(double) | 1.403 | 31.42 | 0.9505 | 0.154 | 43.30 | 0.9602 | 0.002 | 46.81 | 0.0456 |
| CCB650 | Cd(Single) | 1.694 | 8.17 | 0.9769 | 0.109 | 25.52 | 0.9748 | 0.019 | 27.88 | 0.9503 |
|  | Pb(Single) | 2.149 | 20.21 | 0.9820 | 0.304 | 37.38 | 0.9987 | -0.007 | 44.59 | 0.5063 |
|  | Cd(double) | 0.378 | 10.56 | 0.9499 | 0.002 | 14.54 | 0.9428 | 0.009 | 14.73 | 0.9403 |
|  | Pb(double) | 2.833 | 9.65 | 0.9709 | 0.372 | 33.65 | 0.9900 | -0.006 | 41.69 | 0.6759 |

**
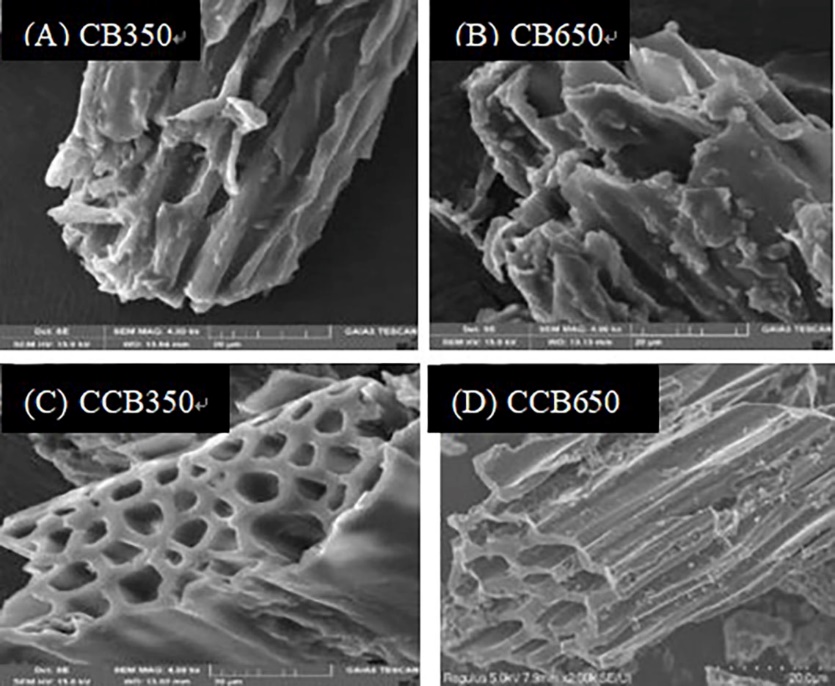
**

**Fig. S1** The SEM images of fresh and aged biochars (A) CB350, (B) CB650, (C) CCB350, (D) CCB650.

**
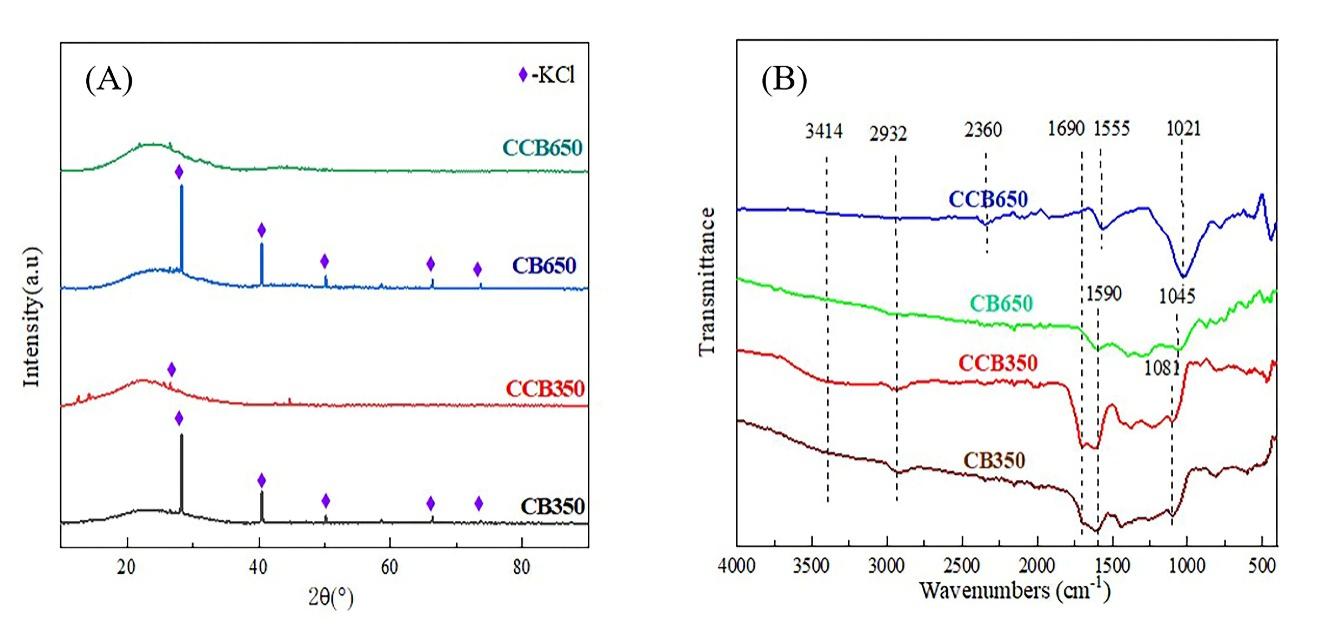
Fig. S2** XRD pattern and FTIR spectroscopy of fresh and aged biochars (A) XRD pattern, (B) FTIR spectroscopy.

CB350

CCB350

CCB650

CB650

**Fig. S3** XPS analysis of the C1s binding energy of fresh and aged biochars.

**Fig. S4** Intra-particle diffusion kinetics of Pb^2+^ and Cd^2+^ on the fresh and aged biochars in the single and binary systems.
